# 3D Mandibular Superimposition: Comparison of Regions of Reference for Voxel-Based Registration

**DOI:** 10.1371/journal.pone.0157625

**Published:** 2016-06-23

**Authors:** Antonio Carlos de Oliveira Ruellas, Marilia Sayako Yatabe, Bernardo Quiroga Souki, Erika Benavides, Tung Nguyen, Ronir Raggio Luiz, Lorenzo Franchi, Lucia Helena Soares Cevidanes

**Affiliations:** 1 School of Dentistry, Federal University of Rio de Janeiro, Rio de Janeiro, Brazil; 2 Bauru Dental School, University of São Paulo, Bauru, Brazil; 3 School of Dentistry, Pontifical Catholic University of Minas Gerais, Belo Horizonte, Brazil; 4 School of Dentistry, University of Michigan, Ann Arbor, MI, United States of America; 5 School of Dentistry, University of North Carolina, Chapel Hill, NC, United States of America; 6 Institute of Public Health Studies (IESC), Federal University of Rio de Janeiro (UFRJ), Rio de Janeiro, Brazil; 7 Department of Surgery and Translational Medicine, University of Florence, Florence, Italy; Medical University of South Carolina, UNITED STATES

## Abstract

**Introduction:**

The aim was to evaluate three regions of reference (Björk, Modified Björk and mandibular Body) for mandibular registration testing them in a patients’ CBCT sample.

**Methods:**

Mandibular 3D volumetric label maps were built from CBCTs taken before (T1) and after treatment (T2) in a sample of 16 growing subjects and labeled with eight landmarks. Registrations of T1 and T2 images relative to the different regions of reference were performed, and 3D surface models were generated. Seven mandibular dimensions were measured separately for each time-point (T1 and T2) in relation to a stable reference structure (lingual cortical of symphysis), and the T2-T1 differences were calculated. These differences were compared to differences measured between the superimposed T2 (generated from different regions of reference: Björk, Modified Björk and Mandibular Body) over T1 surface models. ICC and the Bland-Altman method tested the agreement of the changes obtained by nonsuperimposition measurements from the patients’ sample, and changes between the overlapped surfaces after registration using the different regions of reference.

**Results:**

The Björk region of reference (or mask) did work properly only in 2 of 16 patients. Evaluating the two other masks (Modified Björk and Mandibular body) on patients’ scans registration, the concordance and agreement of the changes obtained from superimpositions (registered T2 over T1) compared to results obtained from non superimposed T1 and T2 separately, indicated that Mandibular Body mask displayed more consistent results.

**Conclusions:**

The mandibular body mask (mandible without teeth, alveolar bone, rami and condyles) is a reliable reference for 3D regional registration.

## Introduction

Broadbent[1], in 1931, described a technique that made possible the longitudinal study of facial growth through a standardized and reproducible method. Despite some disadvantages of this method, such as small magnification, distortion, and head positioning errors, superimpositions have been accepted for clinical and research purposes in orthodontics.

Variations in direction and intensity of growth, rotations resulting from the growth and tooth eruption can be evaluated in longitudinal studies. Serial cephalometric radiographs[2–4] have been used for dynamic studies of dental and skeletal changes in growing children. Longitudinal implant studies have indicated stable areas of reference for understanding regional changes during growth.[2, 5, 6] Superimposition method using implants[7, 8] made it possible to discern between displacement and sites of remodeling (bone apposition and resorption) of the individual jaw.

Superimposition on stable mandibular structures can be used to evaluate growth and treatment changes in the mandible without the use of metallic implants.[9, 10] The structural method[9] of superimposition was based on the following stable structures of the mandible: (1) the anterosuperior contour of the chin; (2) the inner cortical structure of the inferior border of the mandibular symphysis; (3) the trabecular structure in the symphysis; (4) the contour of the mandibular canal; and (5) the lower contour of the developing third molar tooth germ before root development begins.

Cone beam computed tomography (CBCT) can be an effective tool to study growth and treatment results because it provides actual measurements without magnification,[11] allowing image comparison with accuracy and precision;[12] it also enables volumetric measurements of an object and assessment of changes in contour and shape which are often limited in 2D cephalometrics. Besides providing more information than 2D images, 3D images can also be superimposed (or registered), using thousands of points, shapes and volume rather than lines or curves, and allowing for evaluation based on the differences obtained directly from the overlapped images.

One of the goals of 3D superimposition of serial images is to understand how changes in size and shape, and relative spatial positions of skeletal, dental and soft tissue can contribute to a specific skeletal and dental pattern. The 3D surface models can be superimposed for assessing growth, treatment changes, stability, and initial diagnosis.[13] The overlay and the distances between the surfaces from different time points can be used to identify and quantify values and direction of the changes.

Anatomical structures reported to be stable on lateral headfilms for regional superimpositions may not be reliable for 3D analysis that also involves the transverse dimension.[14] Although 3D studies[15, 16] have supported the findings of stability of the symphysis and the mandibular canals in a sagittal view, as reported by Björk and Skieller,[10] it has been shown also that the mandibular canals were relocated laterally during growth.[15] However, the accuracy and reliability of regions of reference are still controversial for 3D images superimposition.

The objective of this study was to evaluate three different regions of reference by comparing the results of 3D mandibular registration to measurements obtained independent of registration methods in a patient’s CBCT sample.

## Material and Methods

This patient’s sample study was approved by the Institutional Review Boards of the Federal University of Rio de Janeiro, Brazil (IRB approval number 931.414). The IRB committee waived the informed consent because the proposed project involved only access to secondary data that could not be linked directly or indirectly to a specific individual.

The patients’ sample consisted of 16 growing subjects (from 9 to 13 years) comprised of patients who had two CBCT scans available at two time points (T1 and T2) taken at least 18 months apart from each other, using a 16x22cm FOV and 0.4 mm voxel size. Images were selected using the following criteria: *Inclusion criteria*: (1) acceptable quality images; (2) sufficient FOV to include basion, porion, glabella and chin. *Exclusion criteria*: (1) motion artifacts; (2) large number of metallic artifacts; (3) presence of orthodontic appliances.

### Image analysis for patients’ sample study

The original DICOM files (baseline) were converted to de-identified files in “gipl.gz” format using ITK-SNAP. 3D image analysis was performed based on the following steps:

#### 1. Construction of the of 3D volumetric label maps (segmentation)[17] of the T1 cranial base, maxilla and mandible

The construction of 3D volumetric label maps (segmentations) of the T1 scans were performed using ITK-SNAP open-source software[18] (http://www.itksnap.org). The semi-automatic segmentation procedures in ITK-SNAP utilize active contour methods to compute anatomic structures based on the CBCT image gray level intensity and boundaries. The threshold was adjusted scan by scan because ITK-SNAP allows for the adjustment of the parameters for automatic detection of intensities and boundaries, as well as allows users to edit contours interactively.[19] Segmentation served two purposes: 1) to define the anatomic region(s) of interest for the software to look at when performing corresponding voxels registration, and 2) to build 3D surface mesh models for quantitative measurements and qualitative overlay evaluations using Slicer v4.4 (an open-source software, http://www.slicer.org).

#### 2. Head orientation

**Fig 1:**
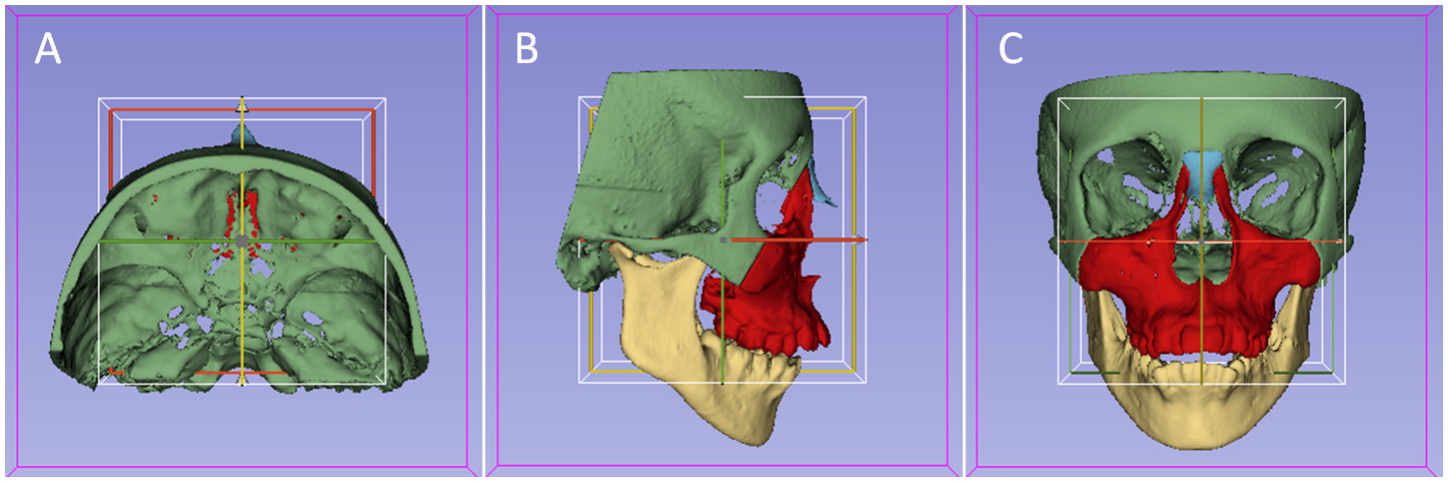
Figures illustrating the head orientation procedure used to obtain the same 3D coordinate system for all patients. **A**) superior view; **B**) lateral view; and **C**) frontal view.

[20] From the T1 3D volumetric label maps, T1 3D surface models were generated and used for head orientation. The T1 3D surface models were oriented using the *transforms* tool in slicer software (Fig 1). The 3D orientation was achieved by using three planes: Frankfort horizontal, midsagittal, and transporionic planes. The midsagittal plane was defined by glabella, crista galli, and basion landmarks. The Frankfort horizontal plane was defined bilaterally by the right and left porion and right and left orbitale landmarks. The transporionic plane was defined bilaterally by porion landmarks that were perpendicular to the Frankfort horizontal plane. Slicer software displayed a fixed 3D coordinate system (the red, yellow and green planes shown in Fig 1) that was used as reference to orient the 3D models. Using axial, coronal, and sagittal views of the 3D models, the T1 model was moved to orient the midsagittal plane vertically, coinciding with the yellow plane of the 3D coordinate system. The Frankfort horizontal plane had to match the red plane. The horizontal transporionic line had to be coincident with the intersection of the red plane and the white box on both sides of the head. The matrix generated from this systematic orientation procedure was applied to the T1 scan and 3D volumetric label map (segmentation), achieving the same head orientation. Then, the samples were oriented in the same 3D coordinate system, obtaining a common head orientation.

#### 3. Approximation of T1 and T2 scans

The T1 and T2 scans were approximated, having as a reference the best fit of the mandible outlines in the 3D multiplanar cross-sections using the transforms tool in Slicer software.

#### 4. Construction of 3D volumetric label maps (segmentation) of the T2 mandible

It was performed using ITK-SNAP software, as described for T1.

#### 5. Placement of landmarks on the T1 and T2 3D volumetric label maps

**Fig 2:**
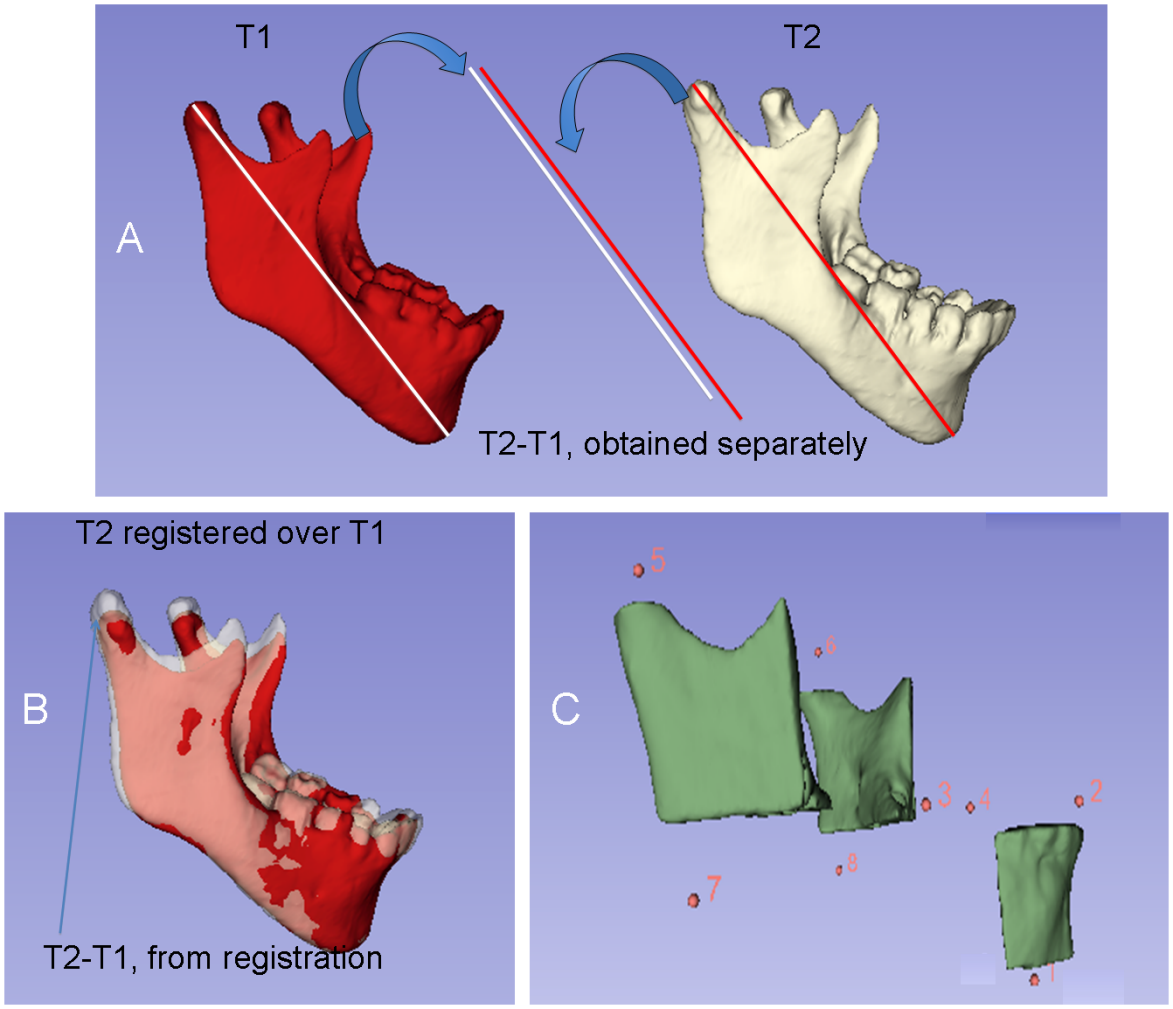
**A**) Representation of the measurements obtained independently from T1 and T2 and their difference; **B**) Representation of the T2-T1 differences based on mandibular registration; **C**) Partial 3D surface model of the mandible and representation of the 8 landmarks used for the measurements.

[21] One label different from the labels used for the 3D volumetric label maps (Fig 2C) was used to indicate landmarks as described in Table 1. Sagittal, axial, and coronal slices, as well as the 3D reconstruction of the image, were used for landmark positioning[22] in ITK-SNAP software. Eight landmarks were pre-labeled[20, 21] in all T1 and T2 volumetric label maps to avoid landmark identification errors after registrations. The landmarks were identified as (Fig 2C): (1) lingual cortex (Cort); (2) lower incisive (Ll); (3) right lower first molar (RL6); (4) left lower first molar (LL6); (5) right condylion (RCo); (6) left condylion (LCo); (7) right gonion (RGo), and, (8) left gonion (LGo). The landmarks were defined on the mandibular surface models oriented in a consistent coordinate system.

**Table 1:** Landmarks identified on patient’s 3D models surface and their definition.

Landmark	Description	Definition
RCo, LCo	right condylion, left condylion	The most superior point of the condylar head, from the posterior and lateral views
RGo, LGo	right gonion, left gonion	The most posterior and inferior point on the angle of the mandible, middle point between buccal and lingual cortical
RL6, LL6	right lower first molar, left lower first molar	Mesiobuccal cusp tip of the mandibular first molar, from the anterior and lateral views
Ll	Lower incisive	Bucco-lingual and mesio-distal midpoint on the incisal border of the mandibular left central incisor
Cort	Lingual cortical	The most posterior point of the inner cortical structure of the mandibular symphysis, from the lateral and inferior views

#### 6. Mandibular voxel-based image registration

**Fig 3:**
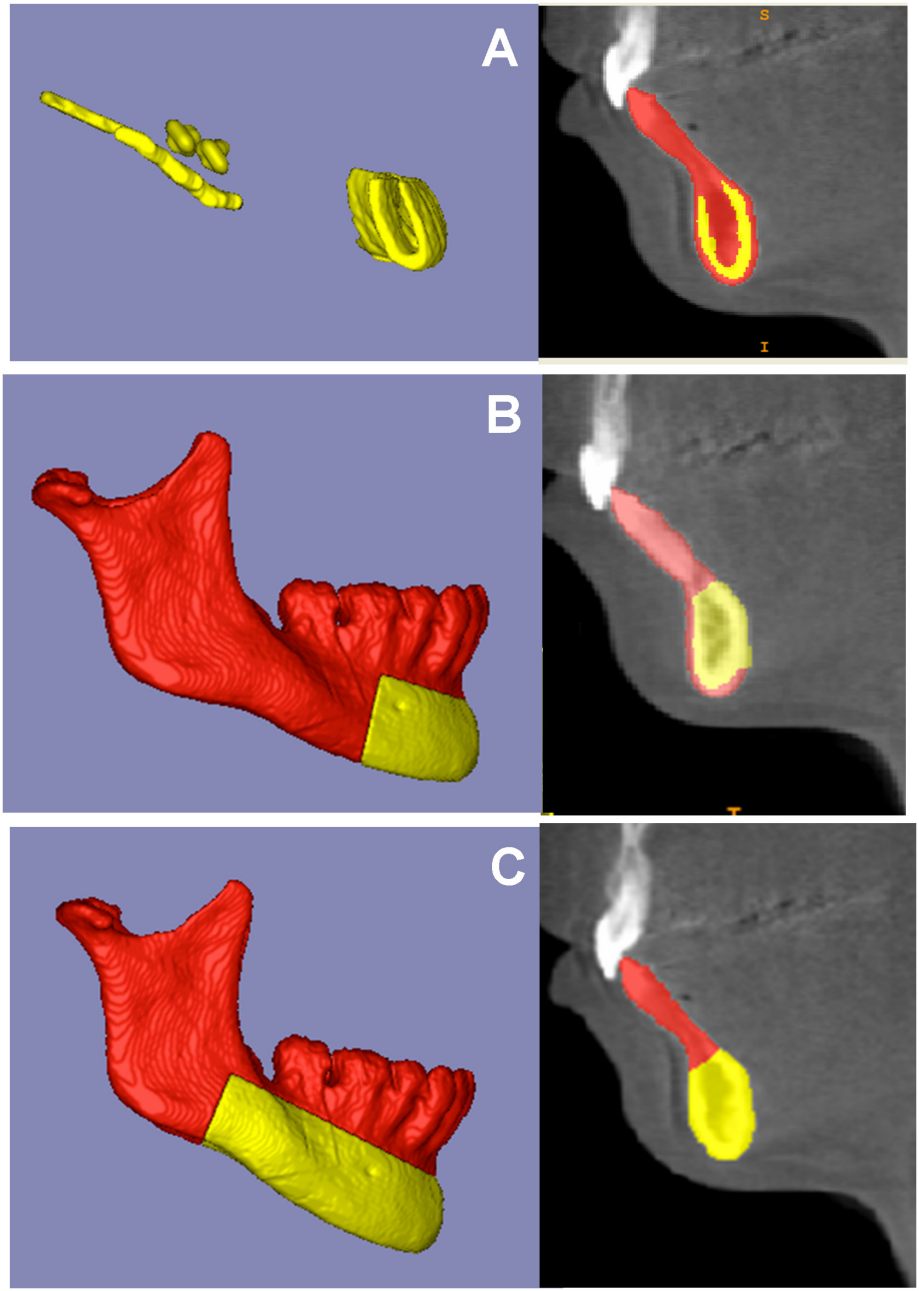
Figures that represent the masks (regions of reference) used for mandibular voxel-based registrations. **A**) Mask1, Björk; **B**) Mask2, Modified Björk; and **C**) Mask3, Mandibular Body. The yellow part corresponds to the mask and the red part was erased.

A fully automated voxel-wise growing registration method[23, 24] was performed using Slicer software. This method used the segmentation of stable anatomic structures as a mask for region of reference, indicating to the software in which areas it should look for corresponding voxels to reach the registration. Three masks were used to register patients’ images: Mask1, or Björk, Mask 2, or Modified Björk, and Mask4, or Mandibular Body (Fig 3A, 3B and 3C). The matrix generated from this step was applied to the T2 3D volumetric label maps (already pre-labeled and approximated) to achieve the same registration in relation to the T1. After registration, T1 and T2 mandibular 3D surface models (.vtk files) were generated for each mask tested. The mandibular registration was used to evaluate 3D spatial differences between T1 and T2, based on their overlapped images.

#### 7. Landmark-based quantitative assessments in Slicer software–quantification of 3D components (Q3DC software tool)

7a) Measurements of distances in T1 and T2 3D surface models independent of registration. The mandibular dimensions were measured separately for each time-point by relating them to the stable reference structure (lingual cortex = Cort), independent of the registration. Linear distances between corresponding coordinates of Cort landmark to each one of others landmarks were measured in each plane of the space (3D components): anteroposterior (y axis), vertical (z axis) and transverse (x axis) distances in T1 and T2 mandibles, independently. The 3D distances (Euclidean distances) between two landmarks were measured as well (Fig 2). The following measurements were used: 1) Cort-RCo; 2) Cort-LCo; 3) Cort-RGo; 4) Cort-LGo; 5) Cort-RL6; 6) Cort-LL6; and, 7) Cort-LI distances. The growth/treatment modifications between two time-points (T2-T1) were evaluated by subtracting the two separate measurements for each time point (independent of the registration).

7b) Measurements of differences between T1 and registered T2 3D surface models based on the voxel-based mandibular registration. By using superimposition methods, the mandibular modifications between two time-points can be quantitatively measured by the difference between the superimposed T2 and T1 surface models. The 3D components between corresponding coordinates of landmarks measured anteroposterior (y axis), vertical (z axis) and transverse (x axis) distances from T1 to registered T2 mandible, and the 3D distance (Euclidean distance) between two landmarks as well (Fig 2). The following measurements were performed between T1 and registered T2 with the Mandibular Body mask as reference and also between T1 and registered T2 using Modified Björk mask as reference: 1) RCo T1 –RCo registered T2; 2) LCo T1 –LCo registered T2; 3) RGo T1 –RGo registered T2; 4) LGo T1 –LGo registered T2; 5) RL6 T1 –RL6 registered T2; 6) LL6 T1 –LL6 registered T2; and, 7) Li T1 –Li registered T2.

The changes observed between T2-T1 (obtained independent of registration–section 7a) were used as “gold standard” and were compared to changes obtained from the two mandibular registrations tested (mandibular body mask and modified Björk mask registrations).

### Statistical analysis

Statistical analyses were carried out with SPSS statistical software package (version 21.0; SPSS, Chicago, IL) and MedCalc (version 14.10.2; MedCalc Software, http://www.medcalc.org).

ICC and Bland Altman tests were applied to compare registrations of patients’ sample by testing concordances of differences between T2-T1 (differences of measurements obtained from each time point separately = nonsuperimposition measurements). These T2-T1 measurements (“gold standard”) were then used to compare to 1) the difference between T2-T1 based on Modified Björk mask registration and 2) the difference between T2-T1 based on Mandibular Body mask registration.

## Results

**Fig 4:**
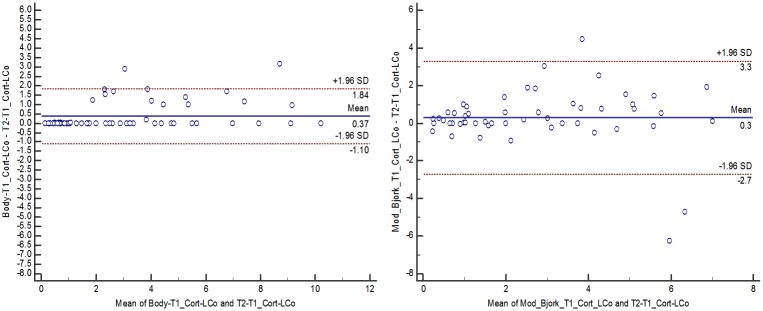
Bland-Altman plots portraying the agreement of changes between T2 and T1 from registrations using Mandibular Body and Modified Björk masks with changes between T2 and T1 obtained from independent measurements (Cort-LCo change, as an example): A) Mandibular Body mask; B) Modified Björk mask. Each circle represents the difference between changes obtained from registrations and changes obtained from independent measurements on T1 and T2. The solid lines indicate the mean difference, and the dashed lines show the 95% limits of agreement (LOA).

When the three masks were applied to register patients’ scans, the software completed the registration process without errors only in 2 cases of 16 using the Björk mask, 14 cases for Modified Björk mask and for 16 cases applying Mandibular Body mask. Then, the Björk mask was not computed for statistical analysis. The concordance and agreement of the changes obtained from superimpositions (registered T2 over T1) compared to results obtained from non superimposed T2 and T1 separately, indicated that Mandibular Body mask displayed more consistent results than Modified Björk mask (Tables 2 and 3, Fig 4).

**Table 2:** Intraclass correlation coefficient (ICC) comparing changes between T2 and T1 from registrations using Mandibular Body and Modified Bjork masks and changes between T2 and T1 obtained from independent measurements. ICC with 95% confidence interval (CI).

	Intraclass correlation coefficient (95% CI)						
	Dif Cort-RCo	Dif Cort-LCo	Dif Cort-RGo	Dif Cort-LGo	Dif Cort-RL6	Dif Cort-LL6	Dif Cort-1
Body	0.98 (0.97–0.99)	0.98 (0.97–0.99)	0.99 (0.98–0.99)	0.99 (0.98–0.99)	0.88 (0.80–0.93)	0.83 (0.72–0.90)	0.98 (0.96–0.99)
Modified Bjork*	0.86 (0.75–0.92)	0.85 (0.74–0.91)	0.89 0.81–0.94)	0.87 (0.77–0.92)	0.62 (0.34–0.78)	0.66 (0.40–0.80)	0.69 (0.46–0.82)

* two cases missed (the registration processes did not complete properly)

**Table 3:** Measurement of the agreement of changes between T2 and T1 from registrations using Mandibular Body and Modified Bjork masks with changes between T2 and T1 obtained from independent measurements. Bland-Altman means (in mm), standard deviation and 95% limits of agreement (LoA, in mm) for comparison between corresponding measurements of the changes between T2 and T1.

	Results from Bland-Altman: mean (in mm) and 95% LoA (in mm)													
	Dif Cort-RCo		Dif Cort-LCo		Dif Cort-RGo		Dif Cort-LGo		Dif Cort-RL6		Dif Cort-LL6		Dif Cort-1	
	mean	LoA	mean	LoA	mean	LoA	mean	LoA	mean	LoA	mean	LoA	mean	LoA
Body	0.38	1.19; 1.94	0.37	1.10; 1.84	0.18	0.63; 0.99	0.10	0.62; 0.81	0.32	0.88; 1.52	0.31	0.95; 1.58	0.10	0.35; 0.54
Modified Bjork*	0.40	2.90; 3.60	0.3	2.70; 3.30	0.6	2.10; 3.20	0.40	2.00; 2.80	0.30	1.30; 2.00	0.30	1.30; 1.90	0.02	1.30; 1.50

* two cases missed (the registration processes did not complete properly)

## Discussion

A significant amount of knowledge about facial growth and orthodontic treatment results comes from tracings and/or 3D image superimpositions. But since some orthodontic patients have 3D images available, it does not make sense to continue performing 2D superimpositions for them because 2D superimpositions are based on the overlay of lines that are supposedly less reliable than surface or voxel-based registrations.

3D images registration based on the correspondence of voxels[18], particularly the cranial base registration, has been used in the last few years to assess overall facial changes relative to the cranial base, along with other methodologies.[25–27] But regional registrations are still controversial. Recent study[28] validated a method of 3D regional superimposition of CBCT scans for non-growing patients. However, our study aimed to validate a method of mandibular regional registration for growing patients.

For cranial base or regional registrations, CBCT scans often have to be approximated to allow proper automated voxel registration, since patients’ scans are commonly taken with different head positioning. Such scans approximation procedures may lead to inter-observer differences, where different operators, using the same patient scans, the same masks of reference for registration, and same software of choice, may or may not achieve proper registration. In this study we started the registration procedures from the same approximated files, modifying only the mask (region of reference). This fact helped to identify the capability of the software to complete the automated registration procedures using each mask as reference, independent of the operator. Then, reliable masks for reference should indicate to the software regions that are able to achieve the registration, besides small distances between the scans.

The condyles and rami were not included as regions of reference (mask) for regional registration because the most important structures responsible for mandibular skeletal asymmetry are the condyles and rami.[29, 30] They are also important because the growth in the length of the mandible in human beings occurs essentially at the condyles, as confirmed by the implant technique.[7, 31] If these structures are not removed from the mask (that indicates the region of reference), they will interfere in the registration procedure.

The registration failed for 87.5% of the cases (14 cases) when Björk mask was used as reference. This may be explained by a small region of reference in this mask that did not provide adequate control of the 3D translation and rotation of the mandible. The high rate of failures could also be the result of displacement of the mandibular canal, symphysis, contour of the chin, and/or 3^rd^ molar crypt as a result of growth. Other difficulties using this mask could be cited, such as: the mandibular canal is difficult to be localized; third molars are not always present and the worldwide rate of third molar agenesis has been found to be 22.63%.[32]

The registration using Modified Björk mask failed in two cases (Table 2) probably because of the size of the region of reference of Modified Björk mask compared to the Mandibular Body mask that may not be enough to match the voxels of T1 and T2 scans and control the 3D translation and rotation for correct overlay of the images.

By using nonsuperimposition methods, the mandibular changes were evaluated by the difference between the two separate measurements[33] (“gold standard”) and they were compared to changes obtained from the difference between the two 3D models (T1 and T2) registered using Mandibular Body and Modified Björk masks (Fig 2).

For the quantification of growth changes (independent of registration), we chose one region considered stable[9, 15] and easily reproducible on the inner contour of the lingual cortical plate of the mandibular symphysis (Cort) to measure mandibular growth (at the condyle and ramus) and dental changes (at the lower molar and incisor) for each time point separately (Fig 2A).

With superimposition methods, using stable and reproducible anatomical structures for registration, the changes in the condyle and mandible between two time-points can be quantitatively assessed[33] (Fig 2B).

The changes obtained from registered 3D models and the “gold standard” showed better agreement and consistency using the Mandibular Body mask than the Modified Björk mask (Fig 4, Tables 2 and 3). For the Mandibular Body mask, only measurements of the lower molar changes showed ICC < 0.98 but it was still good (ICC = 0.88 and 0.83 for right and left molar, respectively). For the Modified Björk mask, all measurements displayed ICC < 0.9 (Table 2). Bland-Altman evaluation showed broader confidence intervals (Table 3 and Fig 4) for the changes obtained from models generated by Modified Björk mask registration, expressing variability of the results, compared to the separated measurements (“gold standard”).

While the Mandibular Body mask presented excellent agreement and consistent results, it still displayed small differences in relation to the “gold standard” that can be explained by the shape of the mandibular angles and the vertical and transverse position of the molars. For these reasons, sometimes the overlay shows symmetrical changes in the condyles or molars, but the differences of changes from the Cort landmark and condylion or molar can show asymmetry. When the measurement between registered surfaces is used, the anatomical variations can be avoided.

The registration techniques allow a comparison of different time points directly from the 3D surface models overlay.[34] The overlay and the distances between the surfaces can also be used to identify and quantify changes. Usually, orthodontics observation and analysis are performed using a scale of millimeters.[35] The visual evaluation of superimpositions requires professional experience and can present some subjectivity. But the agreement and concordance of the changes based on the landmarks are precise and they showed evident differences between the two regions of reference used for registrations of growing patients’ images.

**Fig 5:**
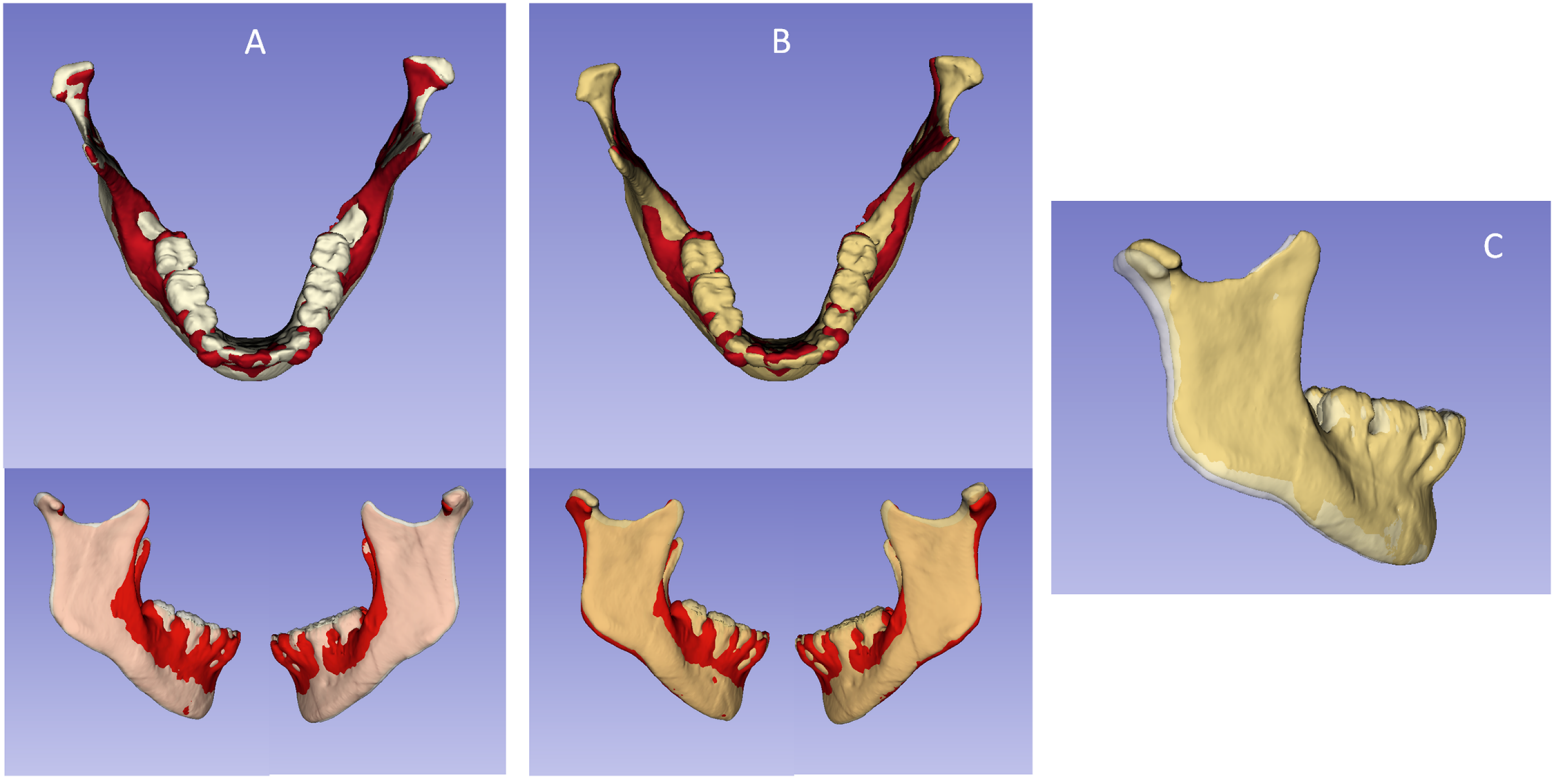
3D surface models overlays: red model, T1; white model, T2 from mandibular body mask registration; yellow model, T2 from modified Björk mask registration. **A**) overlay of registered T2 (mandibular body mask) and T1; **B**) overlay of registered T2 (modified Björk mask) and T1; **C**) overlay of registered T2 (mandibular body mask) and registered T2 (modified Björk mask).

Some overlays of the registered models (using Mandibular Body and Modified Björk masks) over the T1 displayed similar results. Some of them, however, resulted in different interpretations of changes due to growth and/or treatment (Fig 5). It is possible that the Modified Björk mask was not able to control small rotations in the posterior region of the mandible due to its smaller region of reference for registration compared to the Mandibular Body mask.

**Fig 6:**
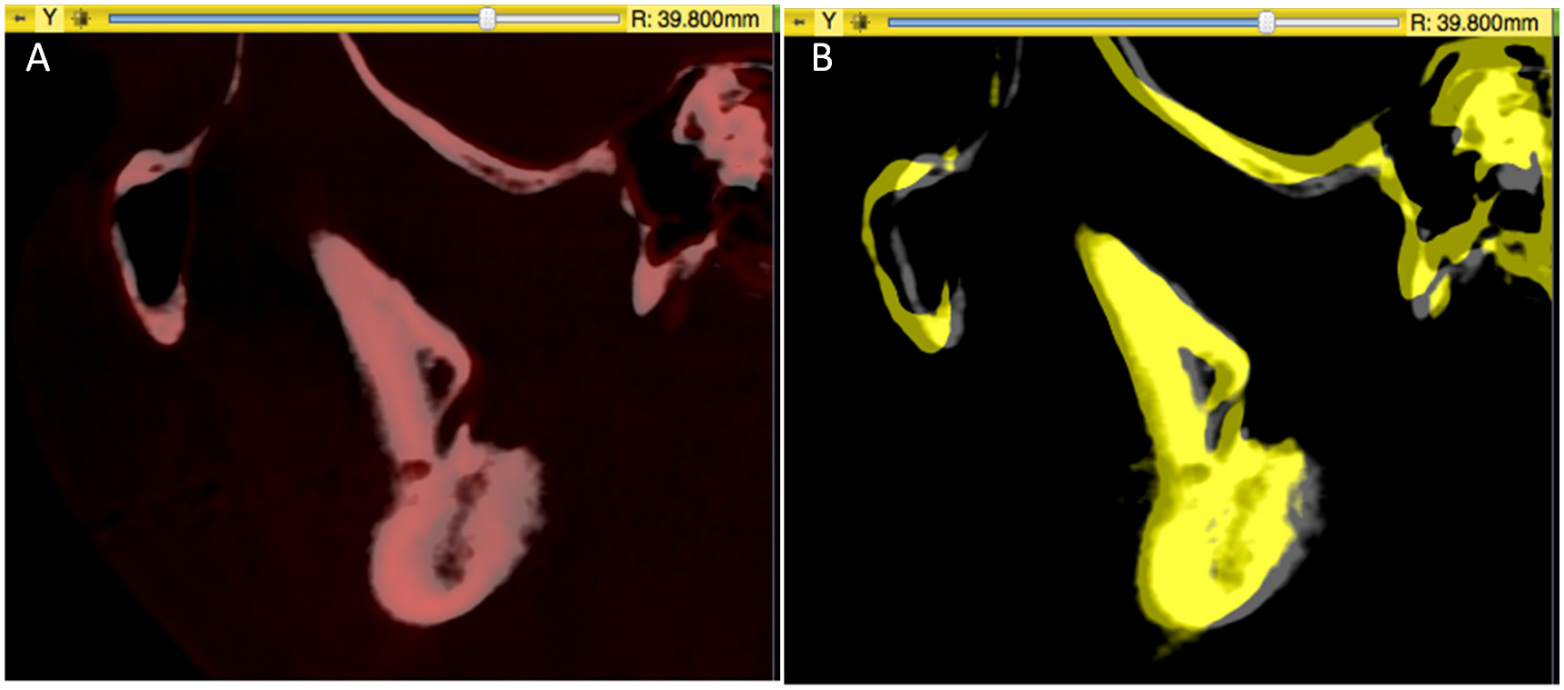
Registered T2 overlapped over T1. **A**) Mandibular Body mask showing superimposition over T1; **B**) Modified Björk mask displayed a vertical shift of the mandibular canal in relation to T1.

Fig 5C shows the evident pitch between the two T2 registered by two different masks (Mandibular Body and Modified Björk). This fact can affect the evaluation of mandibular skeletal symmetry, because the most important structures to assess mandibular asymmetry are the condyle and the ramus.[29, 30] Some 3D studies reported differences in ramus height and body length between both sides in asymmetric mandibles.[36–38] Looking at the patient scans superimpositions (registered T2 over T1) shown in Fig 6, we can observe that for Mandibular Body Mask the mandibular canal were superimposed, confirming findings from implant studies superimposition[7, 31] that advocated stability of the mandibular canal in a lateral view. For Modified Björk mask, the T2 canal displayed a vertical shift in relation to T1.

The findings of the present study emphasize that regional mandibular registration is dependent on the target region of reference that can interfere with the evaluation of the changes due to growth and/or treatment. Defining a stable region of reference allows for correct diagnosis and correct evaluation of growth and treatment results. It can also be used to evaluate bone grafting, tooth movement, mandibular symmetry and condylar resorption. Among the three regions of reference evaluated, the Mandibular Body mask displayed better results compared to the established gold standard.

## Conclusions

Mandibular registration is dependent on the mask used as reference. The mandibular body mask (mandible without teeth, alveolar bone, rami and condyles) is a reliable reference for 3D regional voxel-based registration.
